# Seawater intrusion regulates microbial community structure and functional potential in subterranean estuaries of the Yangtze River

**DOI:** 10.3389/fmicb.2025.1724949

**Published:** 2025-12-08

**Authors:** Yunduo Zhao, Dongsheng Li

**Affiliations:** 1 Key Laboratory of State Forestry Administration on Soil and Water Conservation, Beijing Forestry University, Beijing, China; 2 School of Resources and Environmental Engineering, Ludong University, Yantai, China; 3 State Key Laboratory of Marine Geology, Tongji University, Shanghai, China

**Keywords:** subterranean estuaries, seawater intrusion, microbial community, coastal biogeochemistry, Yangtze estuary

## Abstract

Subterranean estuaries (STE) regulate coastal biogeochemistry through microbial activity. However, responses of microbial community structure and function to seawater intrusion in STE under climate change remain unclear. This study employed isotopic techniques and high-throughput sequencing to analyze the changes in microbial community under seawater intrusion. The results showed that the microbial diversity showed positive correlations with the proportion of seawater (PSW), whereas the abundances of predicted functional genes for nitrogen, phosphorus and sulfur cycling exhibited negative correlations with the PSW. Compared to the layer A (relatively high degree of seawater intrusion), layers B and C (relatively low degree of seawater intrusion) showed declines of 80.4% and 78.1% in Chao index and 19.2% and 15.2% in Shannon index, respectively. Predictive functional profiling further suggested a significant decrease in genes for labile carbon decomposition and a significant increase in genes for recalcitrant carbon decomposition, nitrogen, phosphorus and sulfur cycling in layers B and C. The microbial co-occurrence networks in layer A exhibited more node and high stability, whereas those in layer C were high modularity. Different from the traditional view that salinity is the main environmental filter, we found that the selective pressure of specific ions (such as NH₄^+^, PO₄^2−^, SO₄^2−^) could better explain the variations of the microbial community than the total salinity index in the STE. This revealed the response mechanisms of coastal microbial communities to seawater intrusion and provide a theoretical basis for predicting coastal biogeochemical cycling.

## 1 Introduction

As pivotal interfaces between terrestrial and marine ecosystems, subterranean estuaries (STE) regulate water and solute fluxes to the ocean and drive complex biogeochemical transformations (Richardson et al., 2024). The increasing frequency and extent of seawater incursion—exacerbated by global sea-level rise—are reshaping these STE environments (Du et al., 2025), particularly in the salt-freshwater mixing zone of the intertidal area. These gradients drive a profound reorganization of the microbial community (Chen Y. J. et al., 2022; Zhao et al., 2023; Li D. S. et al., 2025) and establish distinct niches for microorganisms mediating methane and nitrogen transformations (Euler et al., 2023). These saline-freshwater mixing zones sustain unique microbial ecosystems that are highly sensitive to both natural hydrological fluctuations and anthropogenic coastal activities (McCall and Pennings, 2012; Zhao et al., 2023; Li D. S. et al., 2025; Xin et al., 2022). The microbial community structure and key functions like denitrification respond rapidly to salinity shifts and nutrient inputs (Adyasari et al., 2024). Crucially, microbial processes in STE modulate the speciation and flux of chemical elements entering marine systems through submarine groundwater discharge (Ruiz-González et al., 2022). While previous investigations have characterized microbial communities in coastal sediments, groundwater, and nearshore seawater (Hong et al., 2018; Sang et al., 2019; Chen X. et al., 2020), critical knowledge gaps persist regarding the structure and function of microbial community and its responses to seawater intrusion in STE under climate change scenarios.

As fundamental drivers of biogeochemical cycles, microorganisms govern the transformation of carbon, nitrogen, sulfur, and phosphorus (Falkowski et al., 2008; Sokol et al., 2022). In STE, microbial community assembly is primarily governed by deterministic processes involving environmental filtering and species interactions (Zhao et al., 2023). Dynamic environmental shifts (e.g., salinity, ionic composition, dissolved oxygen, and nutrient availability) induced by seawater intrusion and saline-freshwater interactions impose multifaceted selection pressures on microbial community (Adyasari et al., 2019; Zhang et al., 2021; Chen Y. J. et al., 2022; Zhao et al., 2023). Indeed, experimental evidence confirms that salinity is a key determinant of microbial community structure and functional gene abundance in STEs (Zhang et al., 2021; Adyasari et al., 2024). Although elevated salinity is generally associated with reduced microbial diversity (Zhang et al., 2019; Chen H. H. et al., 2022) and destabilized ecological networks (Li et al., 2023; Li M. C. et al., 2024), the phenomenon of enhanced microbial interactions under salinity stress (Zheng et al., 2017; Chen et al., 2023; Li Y. et al., 2025) suggests potential metabolic cooperation strategies. The mechanistic drivers underlying microbial community dynamics in STE have not been fully elucidated, particularly the specific ionic factors that control community structure and function.

Anthropogenic perturbations and climate change—notably saltwater intrusion—are fundamentally altering STE ecosystems (Murray et al., 2022). While microbial biogeography patterns are increasingly documented at global scales (Fierer and Jackson, 2006; Labouyrie et al., 2023), microbial community in STE remains disproportionately understudied. Understanding how seawater intrusion reshapes microbial community structure and function is imperative for predicting ecosystem service trajectories (e.g., carbon sequestration, nitrogen removal) and developing adaptive management strategies for climate-resilient coastal zones. We hypothesize that the structure and function of microbial communities in the STE are reconfigured by seawater intrusion, primarily through the imposition of salinity gradients. To verify this hypothesis, isotopic techniques and high-throughput sequencing were used to analyze the changes in microbial community under varying degrees of seawater intrusion. This study aims to: (i) elucidate the distribution pattern of microbial community in STE, and (ii) clarify the mechanistic links between seawater intrusion and the structure and function of microbial community.

## 2 Materials and methods

### 2.1 Site description and sampling

This study was conducted in the STE of the Yangtze River (32.24°N, 119.40°E), which is a representative subtropical monsoon-dominated coastal system (Liu et al., 2020). The region exhibits a humid subtropical climate with a mean annual temperature of 15.8 °C and mean annual precipitation of 1,149 mm (Cui and Shi, 2012). The study area was maintained vegetation-free to eliminate rhizosphere effects on microbial community. The study area was characterized by a typical irregular semidiurnal tidal regime. The tidal level ranged from 0.25 m to 3.4 m, with a maximum tidal range exceeding 5 m and an average of 3.3 m. The total organic carbon/total nitrogen (TOC/TN) ratios in the shallow sediments of this region ranged from 7.87 to 18.80, with an average value of 11.80.

This study collected pore water (0–8 m depth) from five monitoring transects in March 2023 using a hydraulic drill (37 mm in diameter; Supplementary Figure S1). Prior to sampling, the tubing was purged by continuously pumping water for 3–5 min using a peristaltic pump until discharging more than 1 L of water. Spring tides were strategically selected as the sampling phase due to intensified submarine groundwater discharge (SGD) fluxes, with tidal amplitudes ranging from 0 to 5 m (Li D. S. et al., 2024). A total of 92 pore water samples were collected during flood tide, ebb tide, and slack tide. Seawater samples were obtained by pre-deploying high-density polyethylene (HDPE) bottles below the low-tide line. Groundwater samples were collected from four sites (Supplementary Figure S1). All water samples were immediately filtered on-site through 0.22 μm sterilized membranes (Aquosystem, 50 mm). The filters were stored in 5 mL sterilized centrifuge tubes for subsequent16S rRNA gene sequencing (−4 °C). The filtered water samples were poured into 30 mL and 100 mL HDPE bottles for measurement of stable isotopes (^18^O/^16^O) and physicochemical analyses, and shipped under refrigerated conditions (4 °C) for subsequent analysis.

### 2.2 Measurements of environmental variables

The source of pore water is relatively stable and is mainly influenced by the evaporation and mixing of seawater and freshwater (Li et al., 2024). The stable oxygen isotope (^18^O) was measured as a conservative tracer to quantify the proportion of seawater (PSW) in the pore water samples. This approach is based on the distinct δ^18^O signatures typically exhibited by terrestrial groundwater and seawater. A two-end-member mixing model was applied using the following Equation 1:


(1) $$a\delta^{18}O_{s}+b\delta^{18}O_{f}=\delta^{18}O_{p}$$

where a and b denote the contribution of freshwater and seawater (a + b = 1), and $\delta^{18}O_{s}$ , $\delta^{18}O_{f}$ and $\delta^{18}O_{p}$ represent the values of $\delta^{18}O$ in seawater, freshwater and pore water, respectively. The stable oxygen isotope (^18^O) was analyzed via an isotopic water analyzer (L2140-i, PICARRO).

The STE was vertically divided into three hydrologically distinct layers based on physicochemical properties as well as geological background (specific details are provided in Li D. S. et al., 2024; Li D. S. et al., 2025): (1) the upper saline plume (layer A), (2) the transitional mixing zone (layer B), and (3) the confined sub-silt-clay aquifer (layer C).

Dissolved oxygen (DO), pH, and salinity of pore water were measured *in situ* using a multiparameter water quality sonde (YSI EXO2, United States). Nutrient concentrations (NH₄^+^, NO₃^−^, PO₄^2−^ and SiO₄^2−^) were determined using a continuous flow analyzer (AA3 HR, SEAL Analytical). Dissolved organic carbon (DOC) was analyzed using a total organic carbon/total nitrogen automatic analyzer (Multi N/C 3100, Germany). Dissolved inorganic carbon (DIC) was determined using an isotope ratio mass spectrometer (Gas Bench II-IRMS). Cation concentrations (K^+^, Mn^2+^, Ca^2+^, Mg^2+^) were measured via an inductively coupled plasma optical emission spectrometry (Thermo iCAP 7,600). Anions concentrations (Cl^−^ and SO₄^2−^) were analyzed by ion chromatography (Dionex ICS-5000+). The chemical characteristics of the different layers are presented in Supplementary Figures S3–S12.

### 2.3 16S rRNA amplification and processing of sequencing data

DNA was extracted using the Mag-Bind^®^ Soil DNA Kit (Omega Bio-Tek) with bead-beating homogenization and quantified via Qubit™ 4.0 Fluorometer (Invitrogen, Carlsbad, CA, United States). The V3–V4 regions of bacterial 16S rRNA genes were amplified in triplicate PCRs with primers 341F (CCTACGGGNGGCWGCAG) and 805R (GACTACHVGGGTATCTAATCC) under a touchdown thermal protocol: 94 °C (3 min); 5 cycles of 94 °C (30 s), 45 °C (20 s) and 65 °C (30 s); 20 cycles of 94 °C (20 s), 55 °C (20 s) and 72 °C (30 s); final extension at 72 °C (5 min). The purified amplicons were pooled in equimolar and paired-end sequenced on an Illumina MiSeq platform (Illumina, San Diego, United States).

After sequencing, the two short Illumina readings were assembled via PEAR software (version 0.9.8) according to the overlap and fastq files were processed to generate individual fasta and qual files, which could then be analyzed via standard methods. First, the primer linker sequences were removed, and then the paired reads were merged into a sequence according to the overlap relationship between the PE reads. The samples were identified and distinguished according to the barcode label sequence. Finally, the effective data of each sample were obtained by using PRINSEQ to remove the bases with a tail mass value <20 (set a 10 bp window). In total, 7351153 high-quality sequences were obtained from 92 pore water samples. The sequence number obtained from each pore water sample ranged from 47,175 to 1,33,203. The mean length obtained from each pore water sample ranged from 414.43 to 426.22. The effective tags were clustered into operational taxonomic units (OTUs) of ≥97% similarity using usearch software (version 11.0.667). Bacterial OTU representative sequences were taxonomically classified by BLAST searches against the RDP database. Functional genes for carbon, nitrogen, phosphorus and sulfur cycle were inferred using PICRUSt2 with KEGG Orthology annotations (v2022.05). The datasets presented in this study can be found in online repositories at NCBI (BioProject ID PRJNA989096).

### 2.4 Statistical analyses

The statistical significance of changes in the structure and function of microbial community and the PSW across layers A, B and C of the STE were assessed using independent samples t-tests. The differences of microbial richness in different layers of the TSE were visualized through Venn diagrams, quantifying unique and shared OTUs (“ggVennDiagram” package). Linear discriminant analysis effect size (LEfSe) was employed to identify microbial biomarkers (i.e., taxa that are characteristic and significantly enriched) in different layers of the STE, applying a threshold of LDA score >2.0. Microbial co-occurrence networks were reconstructed using spearman correlations (|*ρ*| > 0.8, *p* < 0.01) and visualized as robustness-validated topological graphs (“microeco” package). The robustness of the co-occurrence networks was evaluated using random node removal (50%) simulations using the “microeco” package. The stability of the “microbial co-occurrence networks was quantified using the negative-to-positive cohesion ratio calculated using the “microeco” package, where a lower ratio indicates higher stability. Non-metric multidimensional scaling (NMDS) based on Bray–Curtis dissimilarity quantified the microbial β-diversity in different layers of STE. The impact mechanism of seawater intrusion (quantified by PSW) on the structure and function of microbial community was analyzed through linear regression (“ggplot2” package) and structural equation modeling with robust maximum likelihood estimation (AMOS v22.0). Statistical analysis was performed with SPSS 13.0 software and R version 4.2.3.

## 3 Results

### 3.1 The structure of microbial community in subterranean estuary

**Figure 1:**
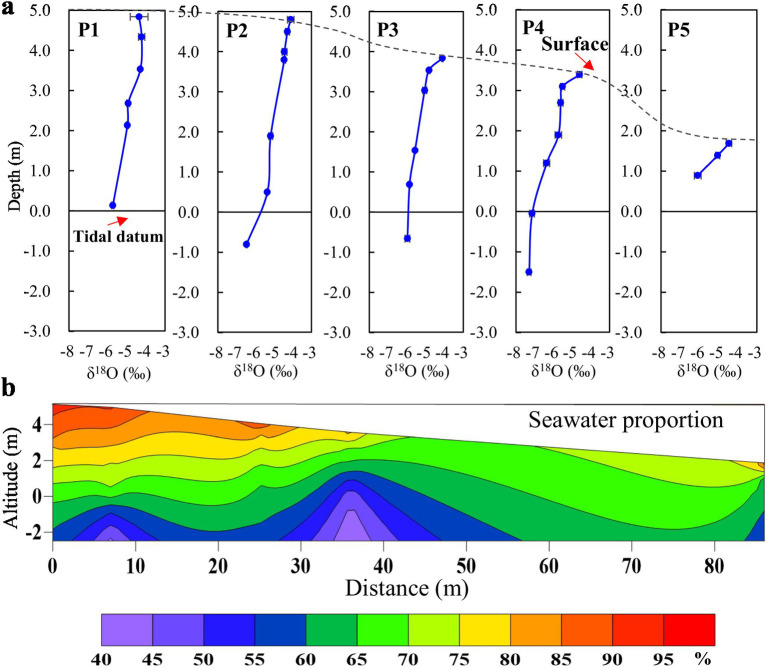
**(a)** Characteristics of stable isotopes in pore water at different layers of the subterranean estuary and **(b)** the proportion of seawater calculated by stable isotopes.

Characteristics of stable isotopes in pore water at different depths and the PSW calculated by stable isotopes are shown in Figure 1. The PSW values were 86.86% in layer A, 75.50% in layer B, and 60.81% in layer C, forming a distinct gradient of seawater influence across the STE. The PSW among different layers (A, B and C) of the STE showed significant differences (*p* < 0.05; Supplementary Figure S2).

**Figure 2:**
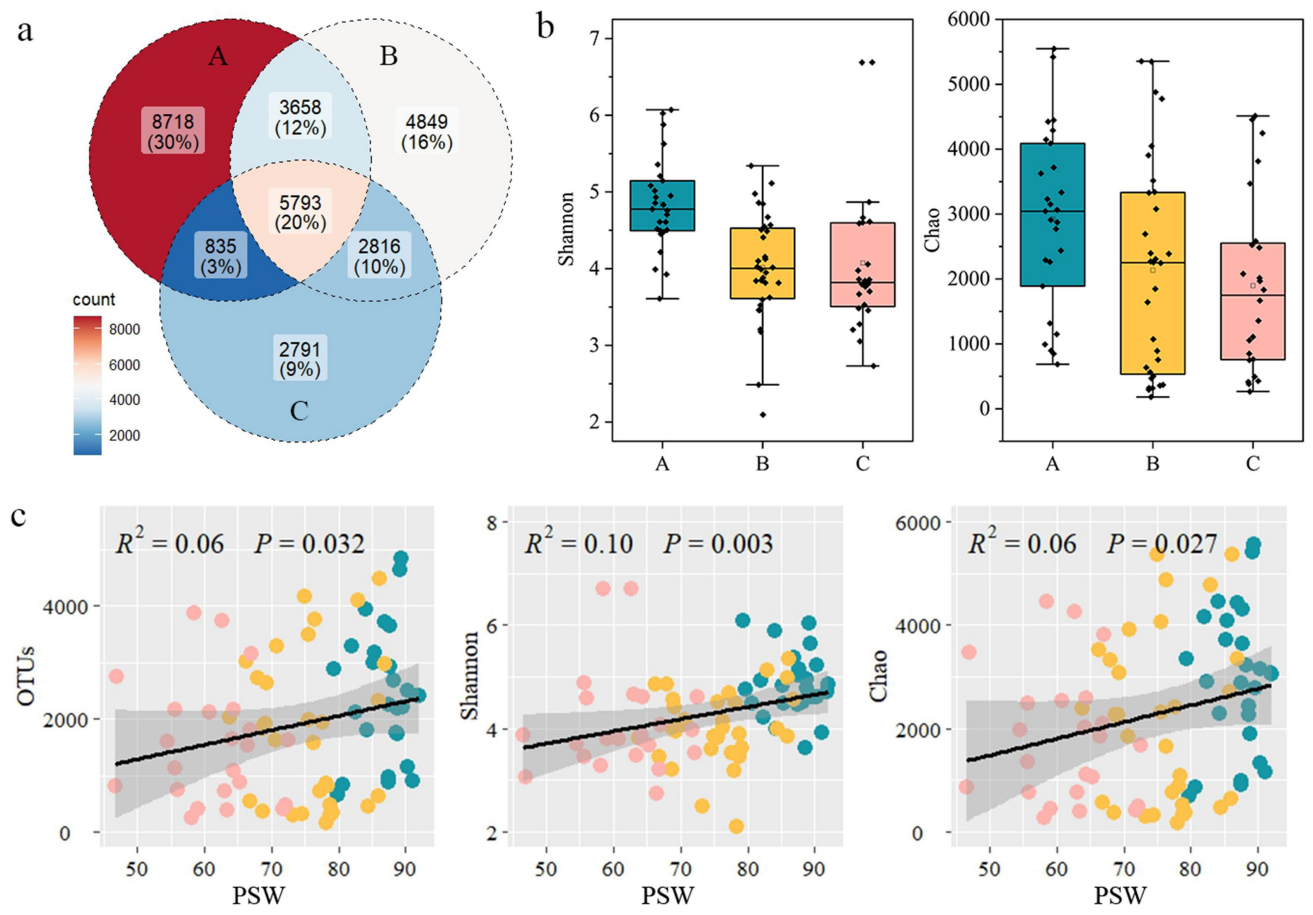
The shared OTUs number **(a)** and microbial α-diversity **(b)** among different layers of the subterranean estuaries. Microbial α-diversity shown as Shannon and Chao indices. **(c)** Relationships between the proportion of seawater (PSW) and the OTUs, Shannon and Chao indices of microbial community. The blue-green, yellow, and pink dots represent data from layers A, B, and C of the subterranean estuary. The shaded area is the 95% confidence interval (CI) of the quadratic regression line.

After sequencing quality control, a total of 29,460 OTUs were identified from all samples. More unique OTUs were found in the layer A of the STE (30%, Figure 2a). The layers B and C showed lower microbial *α*-diversity compared to layer A, with declines of 80.4% and 78.1% in Chao index and 19.2% and 15.2% in Shannon index, respectively (Figure 2b). The microbial α-diversity was related to the PSW in the STE, and showed a positive correlation with the PSW (Figure 2c). In addition, the microbial α-diversity was lower in groundwater than that in seawater (Supplementary Figure S13).

**Figure 3:**
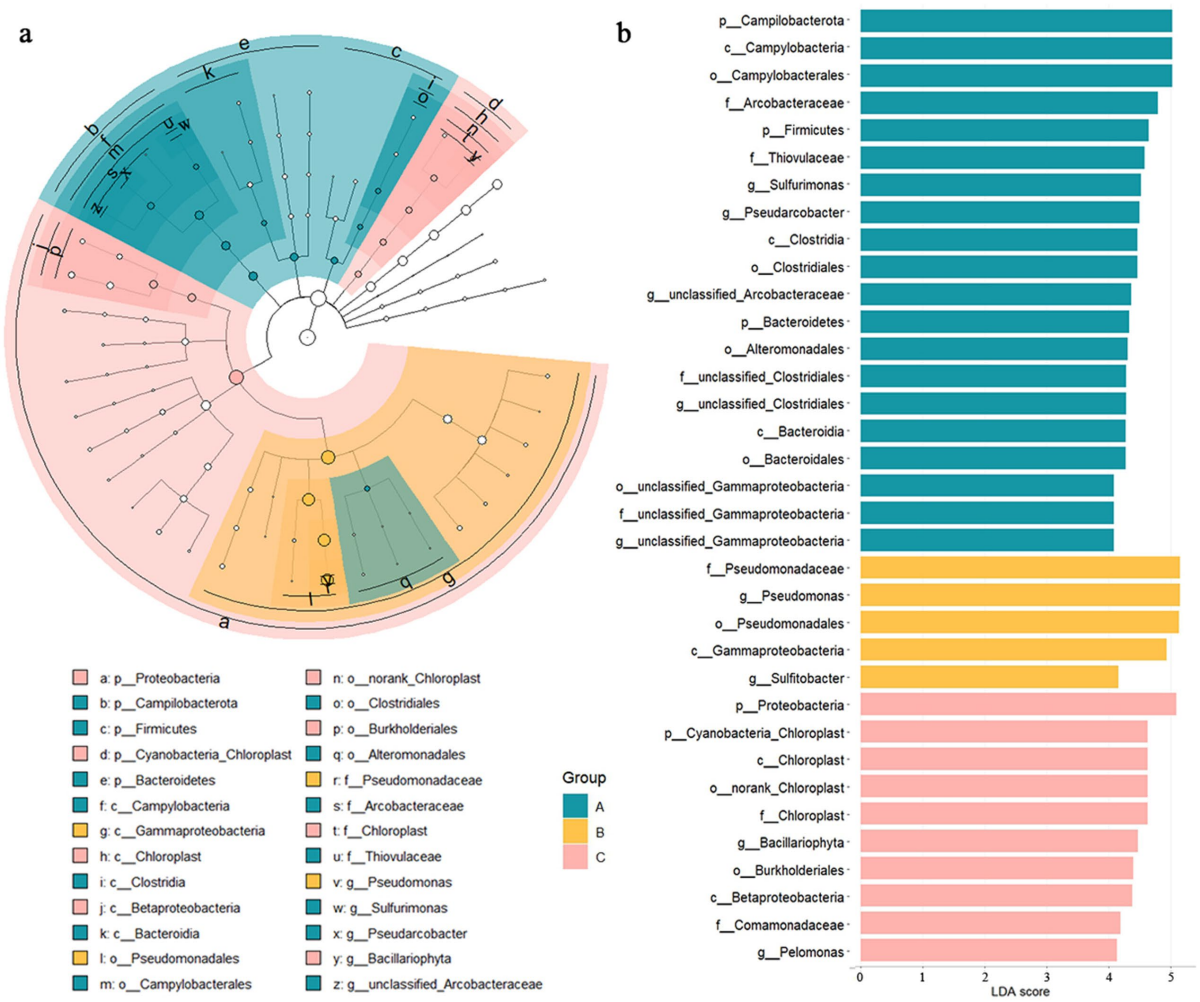
**(a)** Linear discriminant analysis effect size (LEfSe) of microbial community in layers A, B, and C of the subterranean estuary. **(b)** Histogram of linear discriminant analysis (LDA) values among the top 40 biomakers.

The results of LEfSe analysis showed that there were 200, 14 and 47 biomarkers of microbial community in the layers A, B and C of the STE, respectively (Figure 3 and Supplementary Table S1). The biomarkers in layer A predominantly comprised *Arcobacteraceae*, *Thiovulaceae*, *Marinifilaceae*, *Pseudoalteromonadaceae* and *Vibrionaceae*. The biomarkers in layer B included *Pseudomonadaceae*, *Rhodobacteraceae*, *Microbacteriaceae*, *Moraxellaceae*, *Comamonadaceae* and *Chromobacteriaceae*, and the biomarkers in layer C were primarily composed of *Chloroplast*, *Comamonadaceae*, *Burkholderiaceae*, *Bradyrhizobiaceae* and *Rhodobacteraceae*.

The NMDS analysis showed that there were significant differences in the microbial community in different layers of the STE (Supplementary Figure S14). In particular, the microbial community in the layers B and C were significantly different with that in the layer A (*p* < 0.001).

### 3.2 The predicted functional genes in subterranean estuary

**Figure 4:**
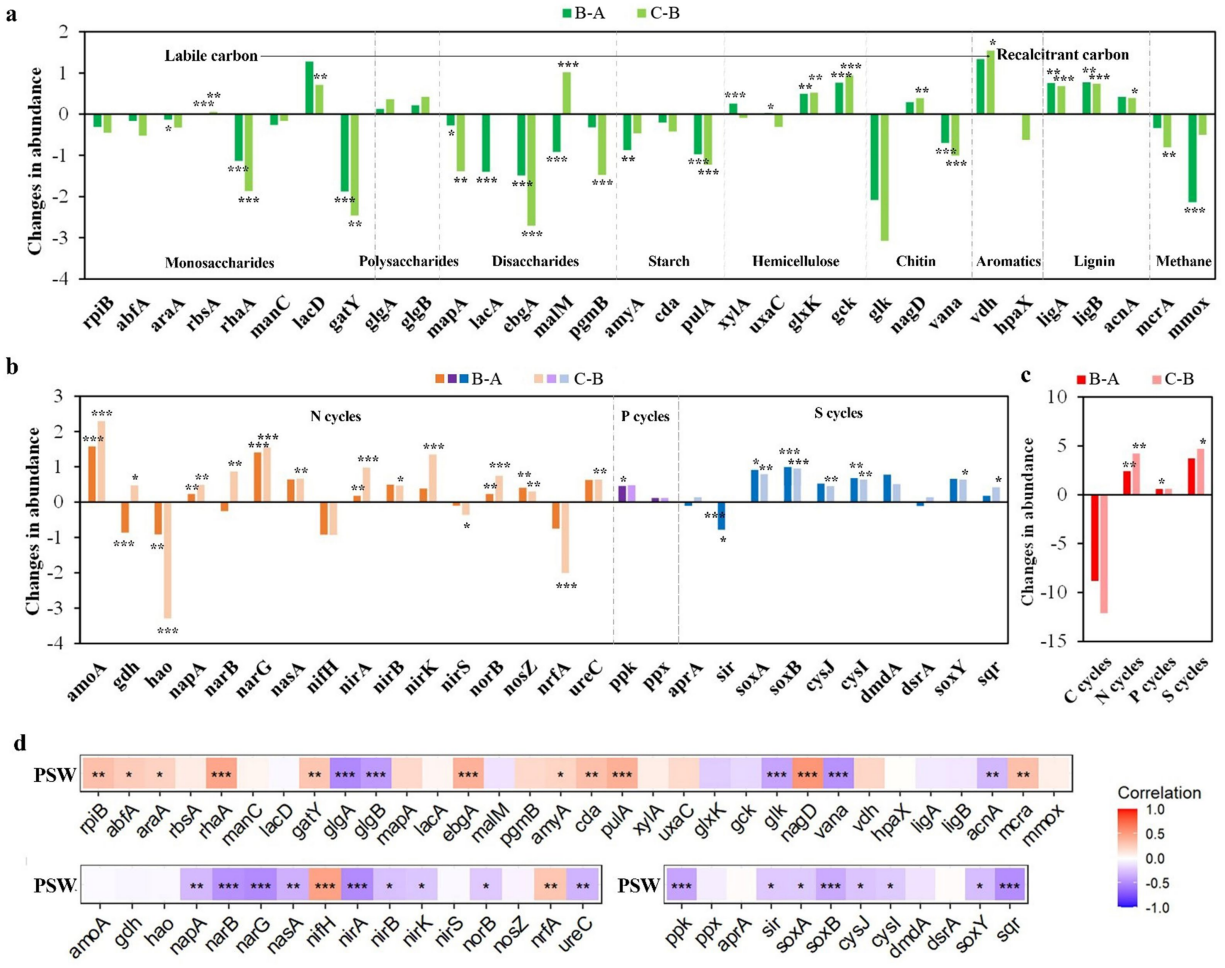
The changes in abundances of predicted functional genes involved in C-cycles **(a)**, N/P/S-cycles **(b)**, and total predicted functional genes **(c)** between layers B, C, and layer A. **(d)** Relationships between the proportion of seawater (PSW) and predicted functional genes based Pearson correlation. * means *p* < 0.05; ** means *p* < 0.01; *** means *p* < 0.001.

Compared with layer A, the microbial functional genes predicted via PICRUSt2 showed significant changes in both layers B and C (Figure 4). Specifically, the abundances of most predicted genes involved in labile carbon decomposition in layers B and C were significantly reduced (*p* < 0.05). In contrast, the abundances of predicted genes associated with recalcitrant carbon decomposition (e.g., *nagD*, *vdh*, *ligAB* and *acnA*) significantly increased in layers B and C (*p* < 0.05). A significant decrease was also observed in the abundances of predicted methane cycling genes (*mcrA* and *mmox*) across both layers B and C (*p* < 0.01).

For nitrogen metabolism, the abundances of predicted denitrification genes (*narG*, *nirk*, *norB* and *nosZ*) and the dissimilatory nitrate reduction to ammonium (DNRA) gene *nirB* were significantly higher in layer C compared to layer A (*p* < 0.05). The abundance of predicted *nrfA* (also associated with DNRA) was significantly lower in layer C than layer A (*p* < 0.001). The abundance of predicted *ppk* (associated with polyphosphate biosynthesis) in layer B significantly increased relative to layer A (*p* < 0.05). Similarly, the abundances of predicted sulfur cycle genes (e.g., *soxA*, *soxB* and *cysI*) were significantly higher in layers B and C than in layer A (*p* < 0.05).

Overall, these results indicate a shift in the predicted functional potential from layer A to layers B and C, characterized by a decrease in the abundances of carbon cycling genes and a concurrent increase in the abundances of genes involved in nitrogen, phosphorus, and sulfur cycling (*p* < 0.05).

The abundances of most predicted genes involved in carbon cycles were positively correlated with the PSW, such as functional genes for decomposing monosaccharides, disaccharide, starch and methanogenesis, while the abundances of predicted genes for decomposing chitin and lignin were negatively correlated with the PSW. The abundances of most predicted genes involved in nitrogen, phosphorus and sulfur were negatively correlated with the PSW (*p* < 0.05), except that the functional genes *nifH* and *nrfA*r.

### 3.3 The microbial co-occurrence networks in subterranean estuary

**Figure 5:**
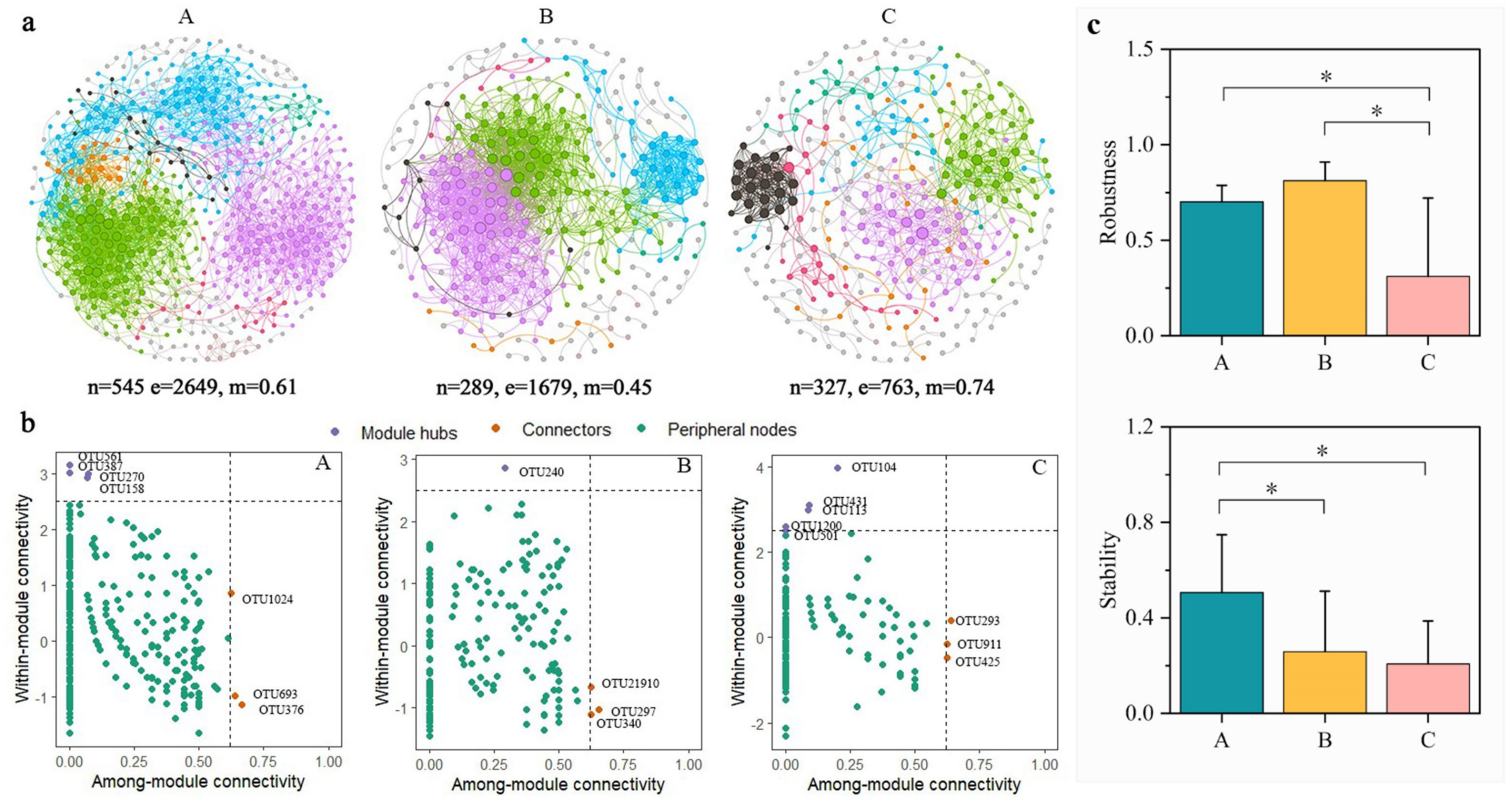
**(a)** Microbial co-occurrence networks in the layers A, B, and C of the subterranean estuary. *n*: Number of nodes; *e*: number of edges; *m*: modularity. Nodes in each network are colored by modularity. **(b)** Zi-Pi plot for the topological roles of bacteria in the layers A, B, and C of the subterranean estuary. **(c)** Robustness and stability of microbial networks in the layers A, B, and C of the intertidal zone. * means p < 0.05.

Microbial co-occurrence networks revealed microbial interaction in the STE (Figure 5a). The number of nodes and edges was higher in layer A than in layers B and C. Although the microbial network size and connectivity decreased, the microbial network in layer C partitioned into more discrete modules, resulting in a significant increase in modularity. This shift toward a more modular architecture is a commonly observed ecological strategy to enhance network stability and resilience under stress. Key taxa (module hubs and connectors) in the co-occurrence network were identified based on the within-module connectivity (Zi) and among-module connectivity (Pi) of each node (Figure 5b and Supplementary Table S2). Layer A had 7 key species of connectors and module hubs (belonging to *Proteobacteria*, *Firmicutes*, and *Fusobacteria*) and layer C contained 8 key species (belonging to *Proteobacteria* and *Aminicenantes*). However, in the layer B, only 4 key species were found, belonging to *Proteobacteria* and *Bacteroidetes*. In summary, the microbial co-occurrence network in layer B has fewer key nodes and the network was simpler.

Based on the robustness and stability analysis of the microbial co-occurrence networks, significant differences were observed among the three layers (Figure 5c). In terms of robustness, both layer A and layer B were significantly higher than layer C (*p* < 0.05). For stability, layer A demonstrated significantly higher values than both layer B and layer C (*p* < 0.05). These results collectively indicate a decline in the stability of the microbial interaction networks from the surface to the deeper layer.

### 3.4 The relationship of seawater intrusion and microbial community in subterranean estuary

**Figure 6:**
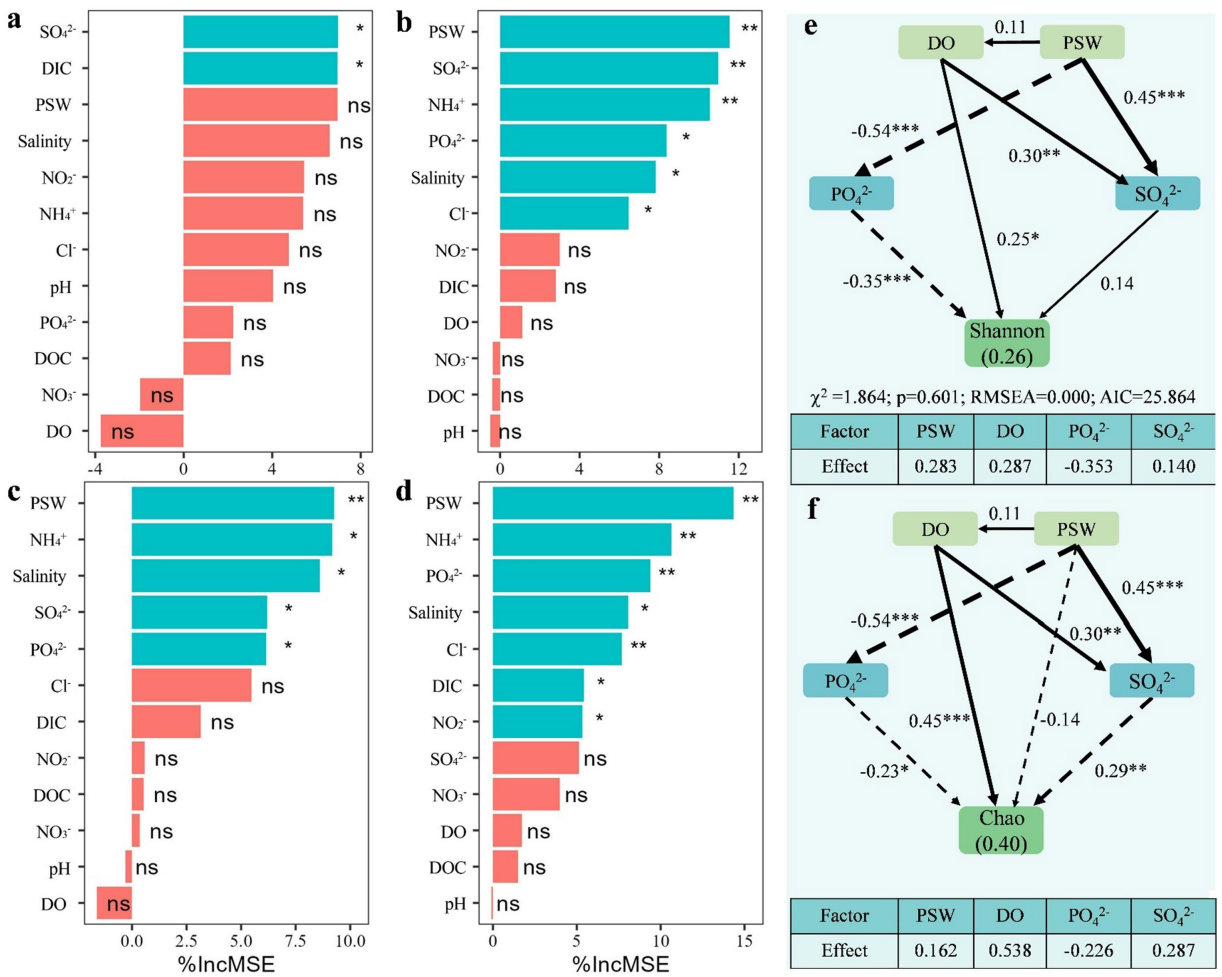
The variable importance (%IncMSE) of factors affecting predicted functional genes (**a**: C cycles; **b**: N cycles; **c**: P cycles; **d**: S cycles) and the impact mechanism changes in microbial diversity in the subterranean estuaries (**e**: Shannon index; **f**: Chao index) based on structural equation model. PSW, proportion of seawater; DO, dissolved oxygen; DOC, dissolved organic carbon; DIC, dissolved inorganic carbon.

The microbial community in the STE was affected by seawater intrusion (Figure 6). The results showed that SO₄^2−^ and DIC emerged as the predominant factors affecting the abundances of predicted functional genes involved in carbon cycles, while PSW was the most substantial influence on the abundances of predicted functional genes involved in nitrogen, phosphorus and sulfur cycles. Ionic gradient modulation (e.g., NH₄^+^, PO₄^2−^ and SO₄^2−^) and salinity also exerted the most substantial influence on the abundances of predicted functional genes involved in nitrogen, phosphorus and sulfur cycles. The Shannon index of microbial community was significantly affected by DO (*p* < 0.05) and PO₄^2−^ (*p* < 0.001), and PSW positively affected the Shannon index through PO₄^2−^ and SO₄^2−^. DO (*p* < 0.001) and SO₄^2−^ (*p* < 0.01) exerted significant positive influence on the Chao index of microbial community, whereas PO₄^2−^ significantly negatively affected the Chao index (*p* < 0.05).

## 4 Discussion

### 4.1 Seawater intrusion altered the microbial diversity

The microbial diversity had significant positive correlations with the PSW. This contradicts the conventional paradigm that high salinity may constrain certain microbial groups through osmotic stress (Zheng et al., 2017) and inhibit microbial diversity (Zheng et al., 2017; Zhang et al., 2019; Chen H. H. et al., 2022). The observed patterns may be linked to the divergent ecological adaptation strategies of different bacteria to environmental gradients such as salinity, redox potential, and nutrient availability: (1) the increase of DO promotes the metabolic activity of aerobic microorganisms and (2) the increased influx of marine-sourced microorganisms increased the proportion of unique OTUs in the layer A. This phenomenon aligns with the finding of higher microbial diversity in higher saline niches (Feng et al., 2023), suggesting that salinity-induced niche stratification in specific habitats may increase microbial diversity.

Further analysis revealed correlations between key ions and microbial diversity: a significant negative correlation with PO₄^2−^ and a positive correlation with SO₄^2−^. PO₄^2−^and SO₄^2−^govern diversity through opposing mechanisms. PO₄^2−^acts as a key environmental factor of marine microbial communities (Deutschmann et al., 2024), which may trigger competitive exclusion by opportunistic taxa. Conversely, SO₄^2−^ acts as an electron acceptor, stimulating a diverse consortium of sulfate-reducers, fermenters, and sulfur-oxidizers (Muyzer and Stams, 2008), which promotes functional niche partitioning and enhances diversity. Consequently, the vertical stratification of microbial community were regulated by multi-dimensional environmental factors including oxygen availability, ionic gradient modulation (e.g., PO₄^2−^ and SO₄^2−^) and species diffusion.

### 4.2 Seawater intrusion modulated the predicted functional genes

The abundances of most predicted genes involved in carbon cycle exhibited highly significant correlations with the PSW. These metabolic strategy shifts were closely coupled with habitat characteristics: (1) seawater intrusion diluted nutrient concentrations thereby driving upregulation of genes abundance for recalcitrant carbon decomposition; (2) declining DO and sulfate concentrations suppressed aerobic mineralization. Therefore, DIC and SO₄^2−^ emerged as the predominant factors regulating predicted microbial genes involved in carbon cycle under seawater intrusion.

The abundances of predicted denitrification genes (*narG*, *nirK*, *nosZ*) and nitrite reducing gene (*nirB*) significantly increased in layer C. The low seawater fraction in this deep layer fostered a hypoxic environment (Supplementary Figure S3), creating a strong selective pressure for anaerobic microorganisms that utilize nitrate and nitrite as terminal electron acceptors. This explains the proliferation of *narG*, *nirK*, and *nosZ* genes. Simultaneously, as nitrate becomes a limiting nutrient (Supplementary Figure S9), the microbial community enhances the *nirB*-mediated nitrite reducing pathway. This represents an efficient nitrogen retention strategy in the deep layers, a phenomenon supported by the superior substrate affinity and electron transfer efficiency of relevant pathways under nitrate limitation (Liao et al., 2025).

Seawater intrusion also altered the abundance of predicted genes related to the phosphorus and sulfur cycling. The diminished inorganic phosphorus content in layer A corresponded to low abundance of predicted *ppk* gene, reflecting adaptations in phosphorus storage strategy. For sulfur cycling, tidal flushing and aerobic decomposition accelerated labile carbon consumption, intensifying competition for O₂ between heterotrophs and sulfur-oxidizing bacteria in the layer A, thereby suppressing sulfur-cycling microorganisms. Sulfur-cycling microorganisms were predominantly affiliated with *Proteobacteria* and *Chloroflexi* (Dombrowski et al., 2018). The negative correlation between the abundance of *Proteobacteria* and the PSW further explained the enriched sulfur-related genes in layers B and C (Supplementary Figure S15).

Notably, salinity exhibited limited effects on predicted functional genes, with nitrogen cycling processes being predominantly regulated by ionic gradients (e.g., NH₄^+^, SO₄^2−^). Sulfur cycling demonstrates critical regulatory roles in microbial biogeochemical processes across riverine, estuarine, and coastal hypoxic environments (Shao et al., 2025). When sulfate serves as the dominant electron acceptor, the accompanying H₂S release during the reduction process can inhibit the biogeochemical cycle of carbon and nitrogen (Callbeck et al., 2021; Shao et al., 2025). In addition, NH₄^+^ is an important factor affecting the bacterial communities in coastal wetlands (Sun et al., 2023), and has a negative impact on microbial diversity (Niu et al., 2022). These findings highlight that seawater interaction may drive changes in microbial functional genes of the STE through modulating habitat heterogeneity, including redox gradients and nutrient dynamics, ultimately shaping spatially stratified metabolic networks in the STE.

This study has limitations in the assessment of microbial function. Although functional predictions were generated from 16S rRNA using PICRUSt2, these findings require validation through metagenomic sequencing, meta transcriptomic, or proteomic analyses to confirm the presence of key genes and their active expression in biogeochemical cycles.

### 4.3 Seawater intrusion regulate the co-occurrence networks of microbial community

Distinct changes in the co-occurrence networks of microbial communities were found in the STE. The networks of microbial communities in layer A (high node connectivity and low modularity) likely arose from tidal fluctuations and diverse organic substrates (Zhao et al., 2023), which induce drastic fluctuations in DO, salinity, and pH, thereby driving extensive microbial interactions to mitigate poly-stress conditions. Notably, seawater intrusion or salinity enhance microbial interdependencies (Zheng et al., 2017; Chen L. et al., 2020). In contrast, the microbial networks in layer B and C exhibited lower node numbers with diminished structural complexity and stability. Microbial communities mitigate resource limitation by forming tightly-knit modules with internal division of complementarity (i.e., niche complementarity). While this strategy may reduce interspecific competition under constant stress, it likely comes at the cost of decreased functional redundancy at the system level. Furthermore, sparse connectivity between modules limits the compensation and dissemination of ecological functions across the network. Thus, the high modularity in layer C can be interpreted as a specialized survival strategy forged in a demanding environment, achieved at the expense of network stability.

While this study provides evidence for the spatial restructuring of pore water microbiota in the STE, two important frontiers for future research remain. First, our sampling captures a snapshot in time; however, temporal factors such as seasonal variations in terrestrial groundwater discharge, temperature, and primary productivity could significantly modulate the patterns. Second, this study focuses on the pore water to elucidate the dynamics of microorganisms in the STE, which are most susceptible to transport via submarine groundwater discharge and seawater intrusion. Sediment-attached microbial communities are also integral to the biogeochemical cycling. Their response to seawater intrusion may differ fundamentally due to their physical association with the sediment matrix. Therefore, future studies that incorporate long-term temporal monitoring and simultaneously analyze both pore water and particle-attached microorganisms will be crucial to develop a predictive, multi-scale understanding of microbial dynamics in these critically reactive coastal ecosystems.

## 5 Conclusion

Our results demonstrate that the reconfiguration of microbial community structure in the STE is attributable to habitat heterogeneity (ionic gradient and DO availability) induced by seawater intrusion, and further suggest this may extend to their functional potential. In zones with a higher proportion of seawater (layer A), microbial communities were characterized by higher diversity, more complex and stable co-occurrence networks, and a greater abundance of predicted genes involved in nitrogen, phosphorus, and sulfur cycling. Specifically, these changes in predicted functional genes were significantly correlated with the PSW. For instance, predicted genes for labile carbon decomposition (e.g., *nagD*, *acnA*) exhibited significant positive correlations with the PSW (*p* < 0.05), whereas predicted genes for nitrogen, phosphorus and sulfur cycling showed significant negative correlations with the PSW. Different from the traditional view that salinity is the main environmental filter, the selective pressure of specific ions (such as NH₄^+^, PO₄^2−^, SO₄^2−^) could better explain the variations of the microbial community than the total salinity index in the STE. Habitat heterogeneity (ionic gradient and DO availability), regulated by seawater intrusion, emerges as the mechanism driving changes in the microbial community. In conclusion, seawater intrusion in the STE is an essential ecological process that profoundly shapes the structure of microbial community and functional potential, and this study can provide valuable insights for the biogeochemical cycles of coastal ecosystems.

## Data Availability

The datasets presented in this study can be found in online repositories. The names of the repository/repositories and accession number(s) can be found in the article/Supplementary material.
