# Supplementary material for: Seawater intrusion regulates microbial community structure and functional potential in subterranean estuaries of the Yangtze River
Source: Front Microbiol. 2025 Dec 8;16:1724949. doi: 10.3389/fmicb.2025.1724949 (PMC12719298; doi:10.3389/fmicb.2025.1724949)
Supplement: Supplementary file 1 [file Data_Sheet_1.docx]

**Seawater Intrusion Regulates Microbial Structure and Function in** **Subterranean Estuaries of the Yangtze River**

Yunduo Zhao ^1^ Dongsheng Li ^2*^

*^1^ Key Laboratory of State Forestry Administration on Soil and Water Conservation, Beijing Forestry University, Beijing 100083, PR China*

*^2^ State Key Laboratory of Marine Geology, Tongji University, Shanghai 200092, PR China*

* Corresponding Author: Dongsheng Li

Tongji University, E-mail: dongshengli7@163.com

Address: State Key Laboratory of Marine Geology, Tongji University, Shanghai 200092, China.


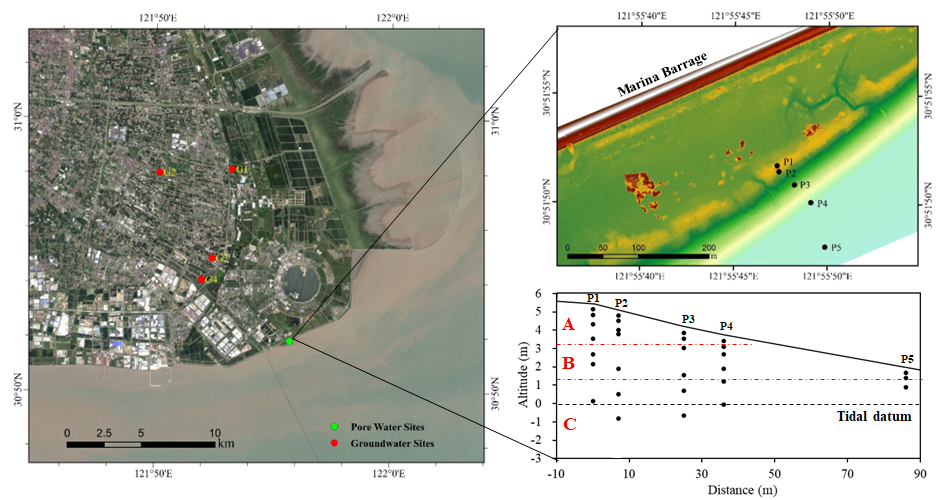


Figure S1 Study area and sampling sites. The tidal datum is defined as 2.67 m below the average sea surface. The subterranean estuary was vertically stratified into three hydrologically distinct layers (A, B and C).
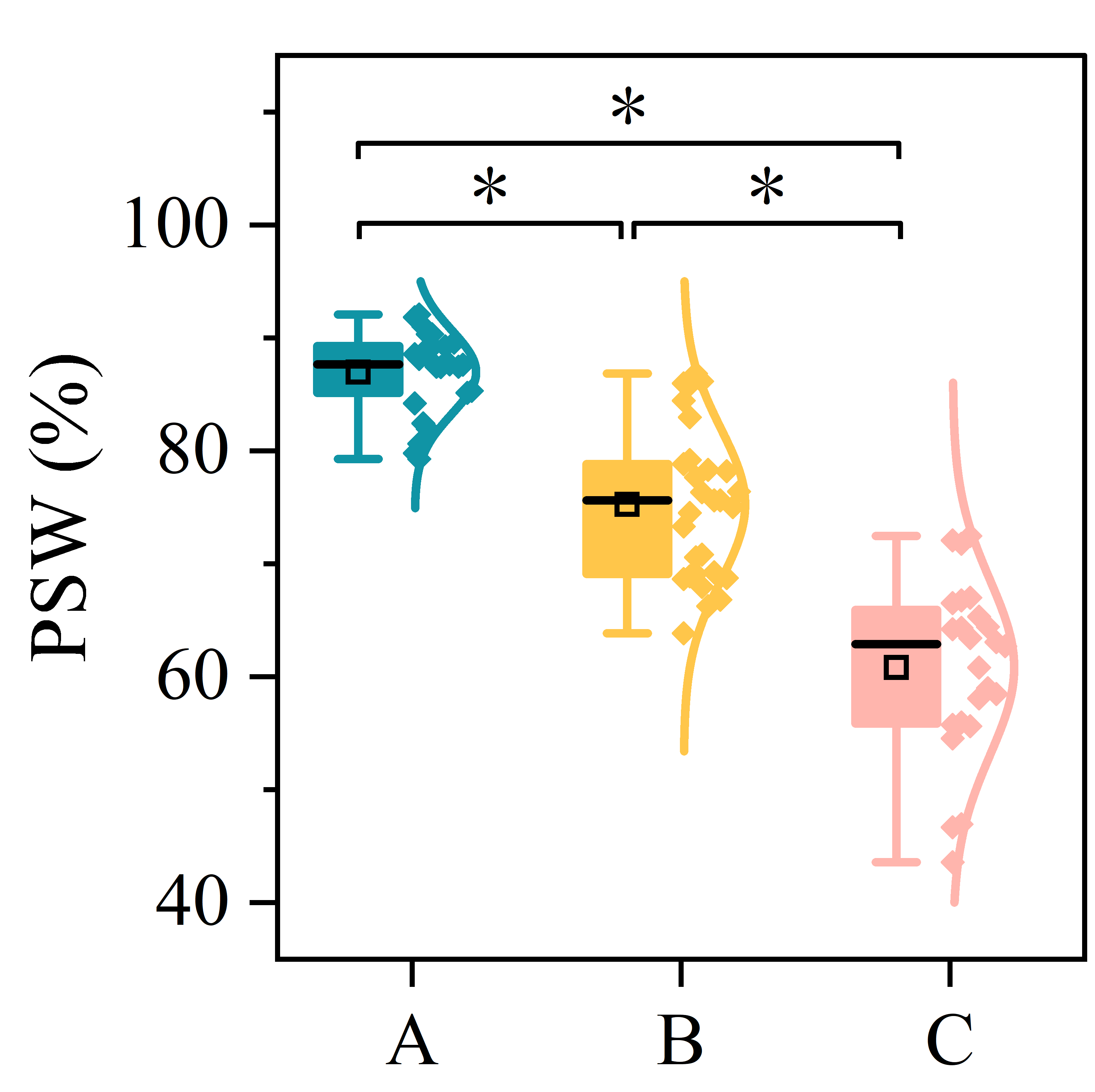


Figure S2 The proportion of seawater (PSW) among different layers (A, B and C) of the subterranean estuaries.

Fig. S3 Values of DO in pore water at different depths.

Fig. S4 Values of salinity in pore water at different depths.

Fig. S5 Contents of pH in pore water at different depths.

Fig. S6 Contents of DOC in pore water at different depths.

Fig. S7 Contents of DIC in pore water at different depths.

Fig. S8 Contents of NH_4_^+^ in pore water at different depths.

Fig. S9 Contents of NO_3_^2-^ in pore water at different depths.

Fig. S10 Contents of NO_2_^-^ in pore water at different depths.

Fig. S11 Contents of PO_4_^2-^ in pore water at different depths.

Fig. S12 Contents of SO_4_^2-^ in pore water at different depths.





Figure S13 The OTUs, Shannon and Chao indices of microbial community in the groundwater and seawater.


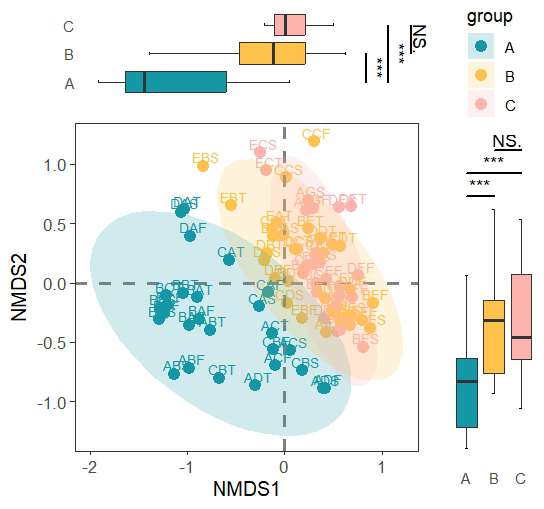


Figure S14 Non-multidimensional scaling (NMDS) ordination of microbial community in layers A, B and C of the subterranean estuaries (Bray-Curtis dissimilarities). *** means p<0.001; NS means p>0.05.


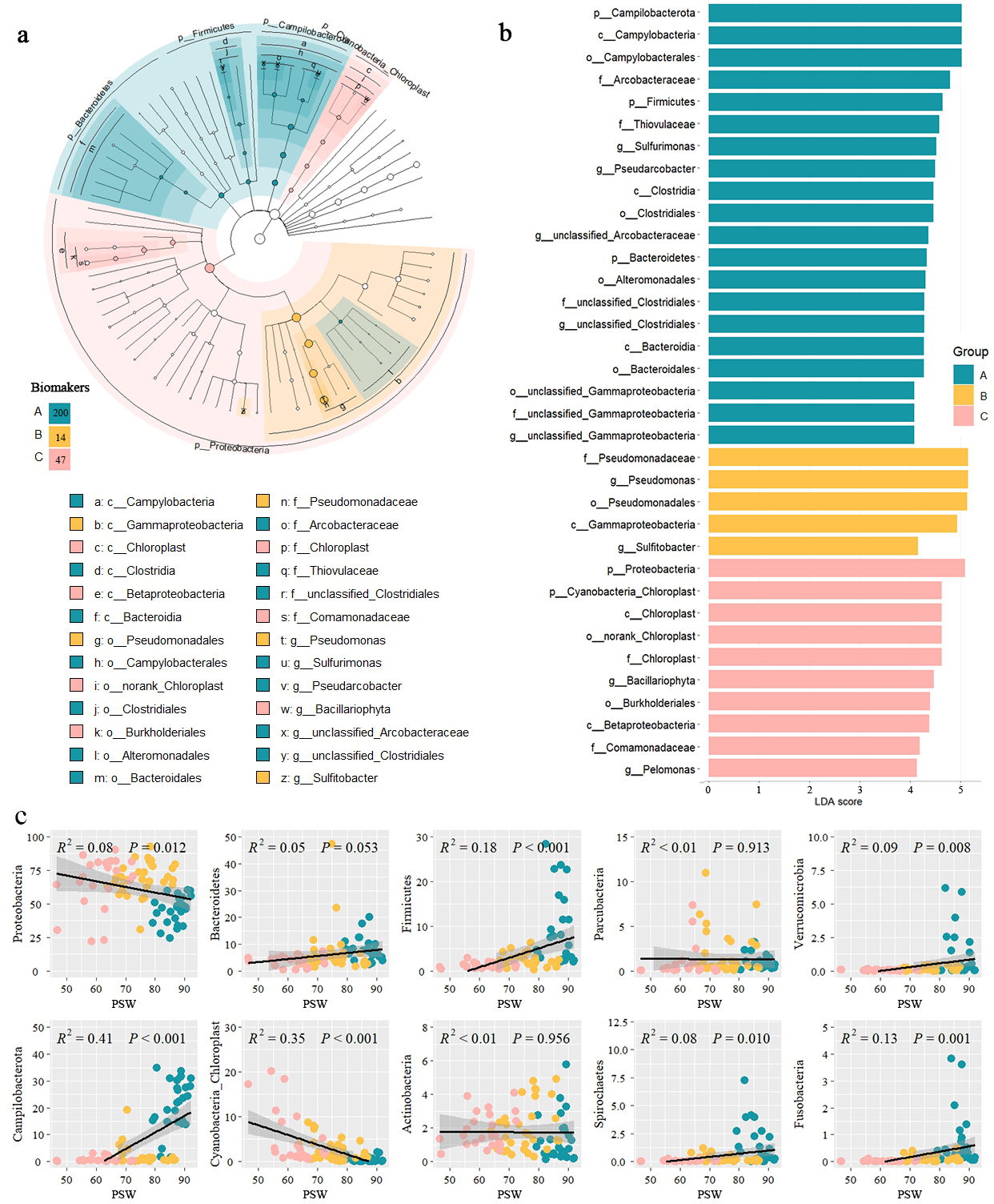


Figure S15 Relationships between the proportion of seawater (PSW) and microbial dominant phyla. The blue-green, yellow and pink dots represent data from layers A, B and C of the subterranean estuary. The shaded area is the 95% confidence interval (CI) of the quadratic regression line.
